# Expression of Reelin, Aβ1-42, Tau and FTH1 in Idiopathic Epiretinal Membranes: Exploring the Link Between Reelin and Neurodegenerative Biomarkers

**DOI:** 10.3390/biom15081187

**Published:** 2025-08-18

**Authors:** Bijorn Omar Balzamino, Graziana Esposito, Pamela Cosimi, Rosanna Squitti, Giuseppina Amadoro, Valentina Latina, Guido Ripandelli, Andrea Cacciamani, Alessandra Micera

**Affiliations:** 1 Research and Development Laboratory for Biochemical, Molecular and Cellular Applications in Ophthalmological Sciences, IRCCS-Fondazione Bietti, 00184 Rome, Italy; bijorn.balzamino@fondazionebietti.it (B.O.B.); graziana.esposito@fondazionebietti.it (G.E.); 2 Retinal Surgery Unit, IRCCS-Fondazione Bietti, 00184 Rome, Italy; pcosimi@gmail.com (P.C.); guido.ripandelli@fondazionebietti.it (G.R.); andrea_cacciamani@hotmail.com (A.C.); 3 Department of Laboratory Science, Research and Development Division, Ospedale Isola Tiberina—Gemelli Isola, 00186 Rome, Italy; rosanna.squitti@fbf-isola.it; 4 Department of Theoretical and Applied Sciences, eCampus University, 22100 Novedrate, Italy; 5 European Brain Research Institute (EBRI), 00161 Rome, Italy; giuseppina.amadoro@ift.cnr.it (G.A.); valentina.latina80@gmail.com (V.L.); 6 Institute of Translational Pharmacology (IFT), National Research Council (CNR), 00133 Rome, Italy

**Keywords:** Reelin, ERM, neurodegeneration, Alzheimer’s disease, ocular fluids, biomarkers, biomolecules

## Abstract

Growing evidence suggests that Reelin signals and cleavages are affected in neurodegenerative diseases, prospecting a potential role for Reelin in the pathogenesis of neurodegenerative processes occurring in insulted retinas. We sought to determine whether Reelin, Aβ1-42, FTH1 and TAU proteins accumulate in ocular fluids of idiopathic epiretinal membrane (iERM) specimens and whether such accumulations depend on disease severity. Comparisons and correlation studies were used to verify the hypothesis of a Reelin, Aβ1-42, TAU and FTH1 marker expressions in this vitreoretinal disease, extending the knowledge on the pathological spectrum of neurodegenerative eye diseases. Aqueous, vitreous and peeled-off ERM samples were collected from patients who had undergone vitrectomy and grouped according to disease severity. We found out that Reelin and Aβ1-42 were expressed in ocular fluids and affected ERMs depending on disease severity. At stage 3, higher Reelin and Aβ1-42 immunofluorescence staining was detected in ERMs, in agreement with the higher Reelin, Aβ1-42, FTH1 and TAU transcript expressions by RT-PCR. Differential expressions of transcripts specific to Aβ1-42, FTH1, GFAP and TAU occurred in vitreal hyalocytes and astrocytes, which selectively responded to vitreal exposure. This is the first study reporting the association between Reelin and ERM disease, highlighting the potential role of Reelin in neurodegenerating and Drusen-affected retinas. The potential association of neurodegenerative mediators with ERM would suggest that part of the neuronal damage activated at the vitreoretinal interphase might be driven by Reelin.

## 1. Introduction

Reelin pathways and protein cleavages are disrupted in neurodegenerative diseases, prospecting a potential role for this glycoprotein in the development of drugs specifically for counteracting the neuronal damage [1]. Reelin signals originate from a specific binding to apolipoprotein E receptor 2 (ApoER2), and a very low density lipoprotein receptor (VLDLR) [2]. Reelin plays a pivotal role in neurogenesis, neuronal networking, synaptic plasticity and neurodegenerative disorders, including Alzheimer’s disease (AD) [1,3,4]. Changes in Reelin levels have been monitored in adulthood, high in younger and low in older brains, and the inflammatory mediated-Reelin impairments seem to be associated with the onset and progression of neurodegenerative disorders [4]. Growing evidence sustains the interaction between Reelin signal components and the few molecules related to AD, Parkinson’s disease and dementia [5,6]. Several experimental models displayed the interaction of Reelin with deposits of amyloid precursor (APP) and beta-amyloid (Aβ), hyperphosphorylated TAU protein, linkage with the disequilibrium between T-helper and T-reg lymphocytes and finally the indirect association with cellular ferroptosis, hallmarks of neurodegenerative disorders, including AD [5,6,7,8]. In addition to neuronal cells, Reelin can be expressed by endothelial cells and can promote the recruitment of inflammatory cells to sites of inflammation [9,10]. Reelin works in concert with a few neuroinflammatory mediators, participating in acute or chronic insults that lead to neuronal loss (neurodegeneration) and playing a role as neuronal protecting molecules, in concert with other neuroprotective mediators (neurotrophins and growth factors) [8,10,11,12]. The discovery of the existence of attempts of neuronal protection inside tissues allowed us to focus on pathophysiological mechanisms leading to neuronal loss to identify mechanisms able to slow down or prevent neurodegeneration (cellular resilience and selected promotion of self-healing) [13,14,15]. These aspects are particularly evident in neuroretinal networking, where neuronal protection is crucially dependent on the activation of Müller cells, which, in concert with astrocytes and resident microglia, can release neuroprotective mediators to support neuronal cells [9,10,11,12,13,14,15].

In the visual system, Reelin plays a crucial role in retinogenesis, retinal healthiness in adulthood and retinal ageing [1,2,16]. Reactive Müller cells, microglia and astrocytes take part in the physiopathology of the retina, and their inflammatory products such as glial fibrillary acidic protein (GFAP), several interleukins (IL-1β, IL6, IL10) and other metabolites can accumulate in human ocular fluids, as observed in human and in vivo samples [17,18,19,20]. Some vitreoretinal diseases such as macular pucker and age-related macular degeneration (AMD) display Drusen formations (the typical extracellular deposits that accumulate between the retinal pigment epithelium (RPE) and the inner layer of Bruch’s membrane, observed in subjects having a high risk of developing AD) [21]. Drusen formations are composed of lipids, proteins, cellular debris and even APP/Aβ, and are observed in retinas from normal-aged patients and retinopathy-affected ones [21]. As recently reported, Drusen can be associated with an increased risk of decline in cognitive abilities, including dementia and AD [21].

Recent studies on retinal imaging and analysis of ocular fluids have pointed at the possibility of using these non-invasive methods for the early detection of neurodegenerative disorders (AD) due to the strong association between Drusen and neurodegenerative ocular events [17,19]. Out of vitreoretinal diseases, idiopathic epiretinal membranes (iERMs) are diseases of the vitreoretinal interphase characterized by avascular membranes with extracellular matrix components that layer over the retina and promote a long-lasting retraction of the retinal interface depending on the grade of inflammation, providing inflammation, cell damage and release of neuromodulators [22,23,24]. Glial cells, myofibroblasts and infiltrating inflammatory cells are entrapped in the avascular membranous scaffold, exerting a progressive remodeling of the vitreoretinal microenvironment, with obvious problems to visual function [25,26]. Patients mainly refer to visual distortion and blurring that are the result of mechanical traction underneath the retina, resulting in Drusen localized under the retina, reactive gliosis, molecular alterations and inflammation, as well as neuroprotective mediators released in situ and in ocular fluids [22,26]. The continuous mechanical distortion of retina, played by the membrane-based retraction, and the long-lasting cellular activation can also disrupt the synaptic organization, alter axonal transport and trigger early signs of retinal neuronal dysfunction, resembling processes observed in central neurodegenerative diseases [22,23,24,25,26]. Reactive Müller cells, activated astrocytes and sensitized microglia contribute to the local inflammation and promote the release of inflammatory mediators (IL-1β, IL6 and other soluble inflammatory mediators) into the vitreal chamber, which might be detectable in human ocular fluids (vitreous) collected at the time of therapeutic surgery [26]. Inside neurosensory retinas, the impaired retinal homeostasis, due to Muller cell functional failure, can lead to blood–retinal barrier breakdown, impaired iron metabolism and ferroptosis due to the accumulation of ferritin heavy chain (FTH1), another hallmark of neurodegenerative disorders [27,28,29]. Some AD biomarkers, including Reelin, were detected in heavy amounts in ocular fluids, showing correlations with plasma levels, an interesting point for the developing of future tests to quantify ocular fluid biomarkers as diagnostic and prognostic markers of AD, especially for those at risk of ocular and cognitive disease [30,31,32]. The observation that Reelin antagonizes Aβ at neuronal synapses might not exclude the possibility of some alternative therapeutic approaches [33].

Since Reelin expression is important not only for normal retinogenesis and adult retinal function but also under pathological disorders and neurodegenerative states, taking part in reactive gliosis and local neurodegeneration, and since it can be safely accessible in ocular fluids at the time of vitreoretinal surgery, the aims of the present study were (i) to verify whether Reelin, together with Aβ1-42, FTH1 and TAU markers, accumulate in ocular fluids (aqueous, vitreous and peeled-off membranes) of idiopathic ERM specimens, and (ii) to understand whether these accumulations might depend on specific cell subtypes and disease severity. Comparisons and correlations were carried out to assess whether these mediators correlate with disease severity and cellular subtypes. Finally, ocular fluids were tested for their inflammatory abilities to stimulate cultures of retinal astrocytes.

## 2. Materials and Methods

### 2.1. Study Population and ERM Grading

A total of 50 consecutive patients (30F/20M; 74.00 ± 6.35 years old), diagnosed with ERM and selected for therapeutic surgery, were recruited and grouped according to disease severity (*n* = 18/stage 2; *n* = 18/stage 3; *n* = 14/stage 4). Demographic, clinical information and biosamples (aqueous, vitreous and peeled-off membranes) were collected from patients who provided written-informed consent. Anamnesis, funduscopic evaluation and biostrumental analysis (spectral domain optical coherence tomography; Spectralis SD-OCT ver.1.5.12.0; Heidelberg Engineering, Heidelberg, Germany) and ERM grading were carried out at the recruiting visit. The inclusion criteria comprised adult patients diagnosed with ERM and selected for therapeutical vitrectomy [26]. The exclusion criteria encompassed patients with stage 1 ERM; patients receiving anti-vascular endothelial growth factor (VEGF) intravitreal injections or topical anti-glaucoma therapy; subjects undergoing eye surgery in the past or retinal laser therapy in the last 3 months prior to surgery; high intraocular pressure (IOP > 22 mmHg); comorbidities such as systemic neurodegenerative diseases (Alzheimer’s disease or Parkinson’s disease) or local/systemic autoimmune diseases (merely Sjögren syndrome and diabetes), as well as any vascular, degenerative or inflammatory diseases.

### 2.2. Aqueous, Vitreous and ERM Management

Sampling was performed at the time of routine 25-gauge pars plana vitrectomy, according to a standard procedure [25,26]. Aqueous collection occurred at the time of phacoemulsification, while vitreous samples were collected before peeling of ERMs. Both samples were quickly stabilized 4 °C in a biosample transport bag and delivered to the laboratory, according to a standardized operation procedure belonging to the iso9001:2015 certified laboratory.

Pure aqueous and vitreous (250–500 μL) samples were quickly centrifuged at 2000 rpm for 7 min (1–14 microfuge; Sigma, St. Louis, MO, USA) to separate free cells from clarified humors and supplemented with 1 µL protease inhibitors/sample (Pierce, Thermo-fisher Scientific, Waltham, MA, USA) and quickly sonicated (VibraCell; Sonics, Newtown, CT, USA) for sprinkles of residual cells or free nucleic acids (RNA/DNA); a further centrifugation (13,000 rpm/7 min) to remove residual debris was also carried out. Both humors (3 µL) were spectrophotometrically analyzed (Nanodrop; Celbio, EuroClone S.p.A, Milano, Italy) before producing aliquots for biochemical analysis.

Peeled-off ERMs were removed and placed on pretreated glass slides (BDH, Milan, Italy), postfixed with BioFix (BioOptica, Inc., Milano, Italy) and stored until epifluorescence microscopy and molecular analysis was carried out or processed in lysis buffer to simultaneously extract total RNA and proteins (MirVana-PARIS™ RNA and Native Protein Purification Kit; Thermo-fisher Scientific).

Three subgroups were produced according to ERM severity (Govetto’s classification, [25]) as follows: stage 2, ERMs associated with widening of nuclear layer and loss of foveal depression; stage 3, ERMs associated with continuous ectopic inner foveal layers crossing the entire foveal area; stage 4, thick ERMs associated with continuous ectopic inner foveal layers and severe disruption of retinal layers [25]. As introduced before, patients with mild and thin ERMs with the presence of foveal depression, defined as stage 1, were not included in this study as they were not eligible for surgery.

### 2.3. Immunoprecipitation and SDS PAGE Analysis

Magnetic beads (Protein A Magnetic Beads; Thermo Scientific Pierce, Waltham, MA, USA) were used for the immunoprecipitation of specific cells (vitreal hyalocytes or retinal astrocytes) and proteins from ocular fluids were used for affinity binding antibodies under a standardized procedure [34]. Briefly, prewashed beads were conjugated with specific antibodies in PBS-Tween 0.05% (PBST; 50 µL beads and 5 µL of antibody: anti-CD11a (10-210-C100; exBio), anti-GFAP (MAB-94169; ImmunologicalScience, Rome, Italy), anti-mouse APP/Aβ (B4; sc-28365; Santa Cruz, Dallas, USA) and anti-rabbit Reelin (NBP3-13177; Novus Biologicals, Bio-techne, Milan, Italy). An antibody–bead complex was kept at room temperature under gentle orbital shaking; after 30 min the complex was cleaned up with PBST and added to 50 µg of the total protein of aqueous and vitreous samples at different ERM stages for 1 h incubation. Finally, the specific antibody–bead–protein complexes were eluted in 2 × loading buffer (Invitrogen, Massachussets, USA) supplemented with β-mercaptoethanol, boiled (98 °C/5 min) and electrophoresed in 4–20% SDS-PAGE minigels (mini protean; Bio-Rad, California, USA). After separation, gels were stained according to a standard protocol (SYPRO Ruby gel stain; Thermo Fisher, Massachussets, USA) and under a B-BOX Blue Light LED epi-Illuminator (Smobio, Hsinchu City, Taiwan) [32]. Band analysis was performed by using ImageJ v1.43 (free available).

### 2.4. Epifluorescent Analysis on ERMs

Prefixed whole mounted ERMs were briefly equilibrated in phosphate-buffered saline (PBS) (10 mM phosphate buffer and 137 mM NaCl; pH 7.5), blocked/permeabilized with 0.1% bovine serum albumin (BSA)/0.3% Triton X100 in PBS before quenching (10 mM NH4Cl) and probed with the following antibodies: anti-mouse APP/Aβ (B4) (sc-28365; 1/100; Santa Cruz Biotechnology; Dallas, TX, USA) and anti-rabbit Reelin (NBP3-13177; Novus Biologicals; Bio-Techne SRL, Milano, Italy). The specific binding was detected using Cy2/Cy3-conjugated species-specific secondary antibodies (1/500–1/700; Jackson ImmunoResearch Labs., Europe Ltd., Suffolk, UK). Nuclei were counterstained with DAPI (5 µg/mL; Invitrogen-Molecular Probes, Eugene, OR, USA). Acquisitions were carried out using the TE2000U epifluorescence microscope equipped with NIS 4.0 software (Nikon, Tokyo, Japan). Internal control sections were provided by substituting the primary antibody with control irrelevant IgGs (Vector Laboratories, Inc., Burlingame, CA, USA) and were used for channel-series setup (Nikon). Fluorescent signals were quantified by the free available ImageJ v1.43 software (NIH—http://rsb.info.nih.gov/ij/; accessed on 17 april 2025). Digital images and graph plots were assembled by using Adobe Photoshop 2024 (Adobe Systems Inc., San Jose, CA, USA).

### 2.5. RNA, cDNA Synthesis and PCR Amplifications

Total RNA was extracted from ERMs (*n* = 25) according to the Trizol protocol (Fisher Molecular Biology, Rome, Italy) and dissolved in 11 µL RNAse free water (DEPC-treated and autoclaved MilliQ water, Millipore, Waltham, MA, USA). A routine spectrophotometric analysis (1.5 µL total RNA per sample) was carried out for RNA quantification/assessment of quality (Nanodrop N1000). Retro-transcription (100 ng total RNA) was carried out by using the MML-V retro-transcriptase, in the presence of dNTPs and Oligo-dT, all from Fisher Molecular Biology. The protocol of cDNA synthesis was performed in a LifePro Thermal Cycler (EuroClone, Milan, Italy). cDNAs (3 µL/target and 1 µL/referring gene) were amplified using the hot start SYBR green PCR Master Mix (Fisher Molecular Biology) in a Biorad CFX96 Real-Time PCR System (Bio-Rad., Hercules, CA, USA), in parallel with negative controls. Cq values (Illumina) from normalized samples showing one melting curve were run in REST. Changes in gene expression at stages 3 and 4 were provided as log2 expression ratios with respect to stage 2 (referring group), considering the H3 house-keeping gene. Primer pairs were synthesized by Eurofin MWG Genomics (https://eurofinsgenomics.eu/) and are summarized in Table 1.

### 2.6. Cell Cultures

Primary cultures of human retinal astrocytes (Innoprot; Bizkaia, Spain) were expanded in complete Dulbecco’s modified Eagle medium/nutrient mixture F-12 (DMEM/F-12, 10% fetal bovine serum (FBS), 1 mM glutamine and 1% pen/strep mix) according to a standardized procedure. Confluent 48 h serum-starved monolayers were exposed to pathological vitreous samples from each categorized ERM. After 3 days of stimulation, monolayers were washed with Hank’s balanced sodium solution (HBSS, Euroclone, Milan, Italy) and trypsin treated, and single cells were harvested for biomolecular analysis. Sister monolayers were used for microscopical evaluations. Untreated cells were exposed to PBS (vehicle) and managed in parallel to be used as controls.

### 2.7. Statistical Analysis and Integrated Optical Densitometric Analysis

Data are shown as mean ± SD or median ± SD depending on the graphical representation. All analysis were carried out using Prism10.4 software (GraphPad Software Inc., San Diego, CA, USA). To satisfy the assumption of data coming from a Gaussian distribution, row values were analyzed by Kolmogorov–Smirnov (KS) and the Shapiro–Wilk (SW) tests for normality checks. Thereafter, ANOVA analysis was used to compare protein expressions between subgroups, while REST–ANOVA coupled analysis was carried out for identifying significant changes in real time PCR experiments. Correlations were assessed by using the free download available R studio for windows. A rho limit of 0.700 was considered a significant correlation at *p* < 0.05. For integrated optical density (IntDen), the 8-bit TIFF saved digital images (512 × 512 or 1024 × 1024 dpi; *n* = 5 sections/slide; ×40/dry 0.75 DIC M/N2) were subjected to single analysis with ImageJ. IntDen data (mean ± SD/ERM/optic field) were calculated, grouped and subjected to statistical analysis. For molecular analyses, all comparisons were carried out considering stage 2 as control, as no routine control was available for this disease, and significance between groups was set at * *p* < 0.05; ** *p* < 0.01 and *** *p* < 0.001.

## 3. Results

### 3.1. Reelin and Aβ1-42 Are Expressed in Ocular Fluids and iERMs at Different Stages

**Figure 1:**
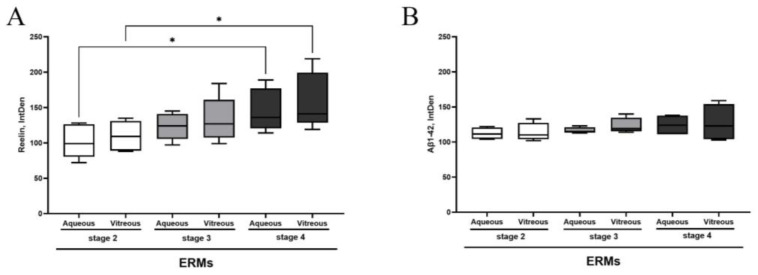
Reelin and Aβ 1-42 protein levels in aqueous and vitreous samples depending on ERM severity. The box plots show the Reelin (**A**) and Aβ 1-42 (**B**) protein expression in comparison between aqueous and vitreous samples at different stages of disease. Asterisks indicate the statistical difference between stage 4 and stage 2, as calculated by ANOVA–Tukey–Kramer post hoc tests (* *p* < 0.05). Legend: ERM—epiretinal membrane; IntDen—integrated density.

As shown in Figure 1A,B, a significant increase in Reelin protein and a trend to an increase in Aβ1-42 protein were observed in both aqueous (Figure 1A) and vitreous samples of ERMs (Figure 1B), with respect to stage 2. Subsequently, we probed ERM tissues with Reelin- and Aβ1-42-specific antibodies to verify the presence of both proteins.

**Figure 2:**
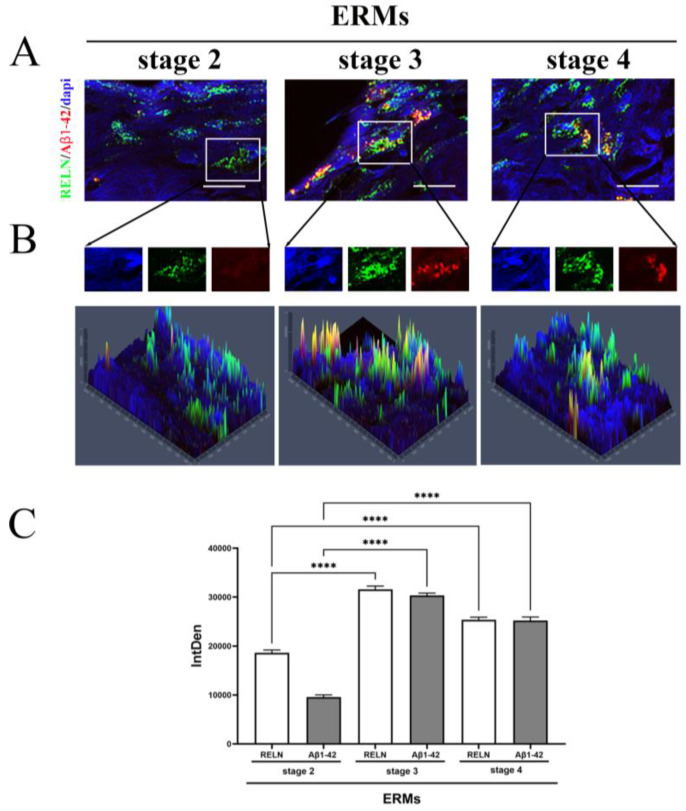
Reelin and Aβ1-42 immunoreactivity in ERMs, by immunofluorescence analysis with quantification by densitometry. (**A**) Representative triple-stained images showing the expression of Reelin (cy2/green) and Aβ1-42 (cy3/red) on nuclear staining (DAPI/blue) (magnification, ×100). (**B**) Representative panels of intracellular localization expressed as pseudo-color carried out by single cell densitometric analysis, starting from random regions (as framed in **A**), with the production of single-channel images. The yellow signal points to a possible co-expression of the two proteins (yellow signal in middle and right panels). (**C**) The quantification of Reelin and Aβ1-42 immunoreactivity in all ERM stages is reported in the IntDen bar graph showing the significant differences by asterisks (**** *p* < 0.0001, ANOVA–Tukey–Kramer post hoc). Legend: ERM—epiretinal membrane; Reelin—RELN; IntDen,—integrated density.

Fluorescent immunolabeled ERMs were subjected to epifluorescence analysis and quantification of fluorescent intensity. As depicted in triple-stained immunolabeled membranes (Figure 2A,B), a trend to an increase in fluorescent signal was monitored for both Reelin (green) and Ab1-42 (red) proteins, depending on disease severity. A widespread Reelin immunoreactivity occurred gradually throughout all stages of disease, while the Aβ1-42 signal started massively at stage 3 and continued at stage 4. An increased immunoreactivity specific for Aβ1-42 (Figure 2B) was monitored at stage 3 and stage 4, particularly at stage 3 and decreasing at stage 4, while almost absent at stage 2. The densitometric analysis (IntDen) is shown in Figure 2C. This high protein expression was in line with the observation of protein expression in vitreal fluids.

### 3.2. Expression of Reelin, Aβ1-42, FTH1 and TAU Transcripts in iERMs

**Figure 3:**
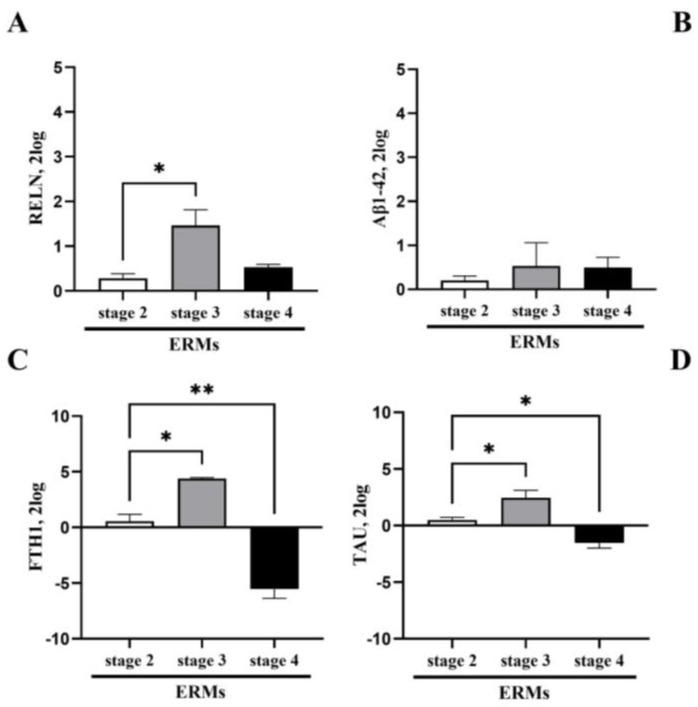
Differential transcript expressions of neurodegenerative markers in ERMs. The transcript expression of Reelin (**A**), Aβ1-42 (**B**), FTH1 (**C**) and TAU (**D**) is shown by bar graphs. Significant changes in transcript expression are pointed by asterisks (* *p* < 0.05 and ** *p* < 0.001; REST–ANOVA–Tukey–Kramer post hoc).

The proteomic analysis of Reelin and Aβ1-42 in both ocular fluids suggested to investigate the origin of these products. Subsequently, a few selected neurodegenerative associated markers between neurons, oligodendroglia, microglia and astroglia were investigated in ERMs. Significant differences among these two transcript expressions were observed at all ERM stages. Consistent with the immunofluorescence data, RT-PCR confirmed a significant upregulation of Reelin transcripts at stage 3 ERMs with respect to stage 2 (*p* < 0.05), while a slight downregulation of Reelin transcripts was detected at stage 4 (Figure 3A). No significant changes were monitored for Aβ1-42 transcripts at all stages of ERM (Figure 3B). Additional two neurodegenerative biomarkers were tested (TAU and FTH1). Of interest, TAU (Figure 3C) and ferritin (FTH1; Figure 3D) displayed the same pattern of expression at ERM staging, increasing at stage 3 (TAU, * *p* < 0.05: stage 3 vs. stage 2; FTH1, ** *p* < 0.05: stage 3 vs. stage 2 ERM) and decreasing at stage 4 (TAU and FTH1: * *p* < 0.05), suggesting a possible involvement in the disease.

### 3.3. Differential Expressions of Transcript Specific for Aβ1-42, FTH1, GFAP and TAU Occurred in Vitreal Cells

**Figure 4:**
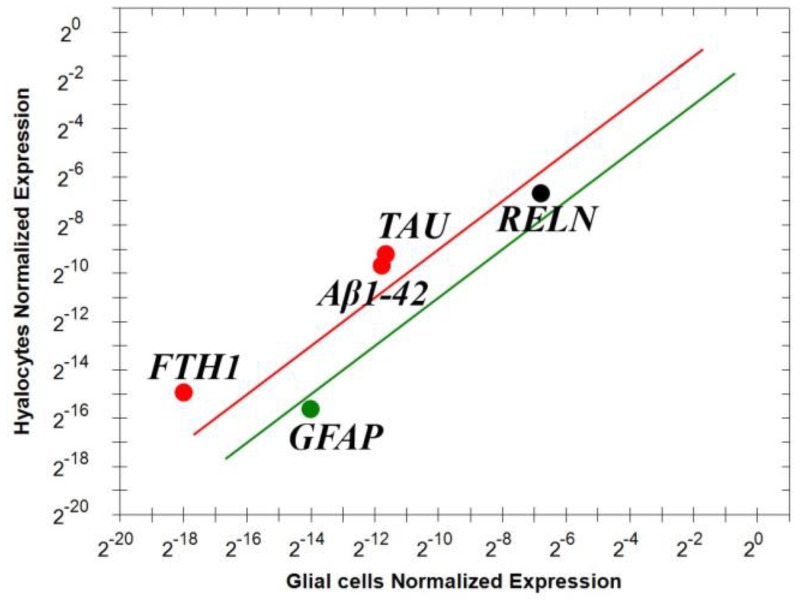
Differential expressions of transcript specific for Reelin, Aβ1-42, FTH1, TAU and GFAP in vitreal cells. Immunoselected vitreal cells were assayed for some specific markers; few selected transcripts (Aβ1-42, FTH1 and TAU). The lines represent the exclusivity of expression, red line for hyalocytes and green for glial cells (*p* < 0.001; REST–ANOVA and multiparametric analysis with Bonferroni correction). Legend: RELN—Reelin; Aβ1-42—amyloid-β 1-42; FTH1—ferritin heavy chain; TAU — TAU protein; GFAP—glial fibrillary acidic protein.

Vitreous cells pelleted from pure vitreous samples and undergoing magnetic bead immune-drive separation were analyzed for real time RT-PCR. To understand the potential origin of these mediators, two phenotypically distinct populations of the vitreous samples, hyalocytes and glial cells (Müller cells, astrocytes and microglia) were obtained and analyzed for Reelin (RELN), GFAP, Aβ1-42, FTH1 and TAU transcript expressions. As shown in Figure 4, the real time RT-PCR showed that these cells contribute differentially to the expression of these transcripts, implying that some out of those markers are solely expressed by hyalocytes (Figure 4). This differential expression highlights the prominent contribution of hyalocytes to the vitreal signature. Indeed, the expression of Aβ1-42 appears mainly by hyalocytes and this is not new as glial cells usually display a more evident GFAP expression.

### 3.4. Astrocytes Selectively Respond to Vitreal Exposure

**Figure 5:**
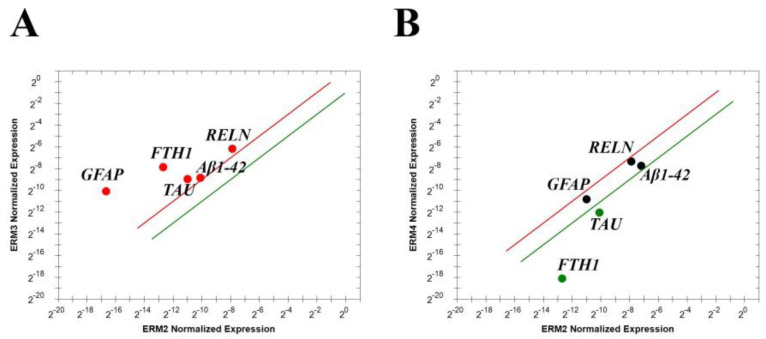
Cultures of normal human astrocytes respond to vitreous exposure. RELN, Aβ1-42, FTH1, TAU and GFAP transcript expressions by ERM vitreal fluid exposed astrocytes. The lines represent the exclusivity of expression for: (**A**) red line for stage 3 and green line for stage 2 and (**B**) red line for stage 4 and green line for stage 2; *p* < 0.001, REST–ANOVA and multiparametric analysis with Bonferroni correction. Legend: RELN—Reelin; Aβ1-42—amyloid β 1-42; FTH1—ferritin heavy chain; TAU—TAU protein; GFAP—glial fibrillary acidic protein.

Since glial cells appeared not to be the major form of vitreal signature, we hypothesized the specific contribution of astrocytes, and, due to the difficulty of separating astrocytes, we tested their potential contribution in vitro. We developed in vitro primary cultures of human normal retinal astrocytes (Innoprot; Bizkaia, Spain). Astrocyte monolayers were exposed to untouched vitreous samples from different stages of disease and, after 24 h, the monolayers were harvested and analyzed for Reelin (RELN), Aβ1-42, FTH1, TAU and GFAP expression by real time RT-PCR. As shown in Figure 5A,B, the exposure to stage 3 and stage 4 vitreous samples triggered a significant increase in Reelin and TAU transcripts by normal astrocytes compared with stage 2 (control). These molecular results suggest that the administration of pathological vitreous samples can directly stimulate astrocytes, causing an overexpression of RELN and FTH1 transcript expressions in a disease severity-dependent manner.

## 4. Discussion

Herein, we confirm the expression of Reelin and Aβ1-42 proteins in human ocular fluids and report for the first time that (i) Reelin and Aβ1-42 proteins are affected in ocular fluids and membranes from patients with ERM, depending on disease severity; (ii) higher Reelin and Aβ1-42 protein expressions occur at stage 3 ERM, as confirmed by higher Reelin, Aβ1-42, FTH1 and TAU transcript expressions at stage 3 ERM; (iii) vitreal hyalocytes participate in the process with increased Aβ1-42, FTH1 and TAU transcript expressions at stage 3 ERM; and, finally, (iv) primary cultures of stage 3 ERM-vitreous exposed astrocytes might participate in the process of overexpression of transcripts specific for Reelin, Aβ1-42, FTH1 and TAU.

Previous studies reported the possibility to quantify some mediators of neurodegeneration in ocular fluids obtained from subjects who underwent pars plana vitrectomy, including biomarkers of cognitive decline and neurodegeneration, as previously reported for experimental models [25,27,30,31,32,35,36]. Herein, three cohorts of biosamples collected from subjects with ERM who had undergone phaco-vitrectomy were examined for their simultaneous expression of Reelin, in concert with Aβ1-42, FTH1 and TAU, also known as candidate hallmarks of cognitive decline (dementia and AD) [8,11,15,37,38]. As known, ERM exerts a long-lasting retraction of the vitreoretinal interphase, producing neurogenic inflammation, which starts a variety of neurodegenerative and neuroprotective tasks played by Müller cells, astrocytes and reactive microglia to protect insulted RGCs [39]. Since the vitreous sample represented the reservoir of inflamed retinas, together with aqueous samples that gathered mediators released from intraocular tissues, we analyzed and compared the protein profiles of aqueous and vitreous samples collected at the time of phaco-vitrectomy to find out potential associations between candidates of neurodegeneration and ERM, to better characterize their expression/production [39,40,41].

First, we observed that the levels of Reelin and Aβ1-42 proteins changed in the ocular fluids, with a consistent increase in Reelin protein in both aqueous and vitreous samples, depending on disease severity. These findings are in line with previous studies showing that both mediators are strongly correlated with the initial dysfunction of neuroretinas and might be safely detected in ocular fluids, providing information even in case of subclinical state [42,43,44]. To support this finding, Reelin, Aβ1-42 and FTH1 alterations have been reported to drive all pathological events observed in retinal degeneration (AMD and aceruloplasminemia), indicating that Reelin impairment and Aβ1-42 and iron toxicity take place in neurodegenerating retinas [28,40,45,46]. The levels of total TAU protein in cerebrospinal fluids from subjects with AD have been established to reflect the intensity of the neurodegeneration [41].

To understand the source of this protein profile, an immunohistochemical localization of Reelin and Aβ1-42 inside the membranes was carried out, observing an increased expression of Reelin and Aβ1-42, as well as an active production of Reelin, Aβ1-42, TAU and FTH1 transcripts, all of which peaked at stage 3 ERM. A specific in situ cell localization was not performed since we know from previous studies that these thin and delicate membranes are populated by Müller cells and their processes, in concert with a plethora of other cells, including astrocytes, hyalocytes, RPE, microglia/macrophages and fibroblasts/myofibroblasts [24,26,42,43,44,45]. Our previous analysis on the characterization of ERM specimens indicated mainly macroglia (Müller cells and astrocytes), microglia, myofibroblasts and some entrapped hyalocytes [24,26,44,45,46]. The higher number of Müller cells (91.7% of specimens) versus astrocytes (66.7%) and the lower number of microglia and macrophages have been also described by others [24,26,45]. While the presence of macroglia is generally accepted in ERM, there is a disagreement concerning the presence of Müller cells and astrocytes, although Müller cells are believed to migrate towards the inner surface of the retina and take actively part in ERM formation [42]. Our study and other studies have also reported that astrocytes, microglia and macrophages are more frequently found in stage 3 than stage 4 and stage 2 ERM, implying a significant “inflammatory” contribution to the membrane activity [24,26,42]. By immunofluorescent analysis and RT-PCR quantification carried out on the same ERMs, we observed that the transcript expression of Reelin fulfilled the protein quantification, while the transcript expression of Aβ1-42 was not aligned with the quantified protein amount. This apparent contrasting data might be in line with the cell subtypes populating the ERMs [24,26,47]. A possible explanation might be that reactive microglia, merely macrophages, can uptake and store Aβ protein for degradation purposes, as prospected in other studies [45,46,47]. Alternatively, the activation of direct/indirect negative feedback might be possible, as observed in previous studies for the production of Ab1-42 by reactive Müller cells and astrocytes [48]. Certainly, the possibility that morphological changes (trans-differentiation) occur due to exposure to inflamed vitreous samples allows difficulty in precisely identifying the subset populating ERMs, and also highlights the participation of myofibroblasts [25,26,27,42].

Discriminating against the source of these mediators represents an additional step in characterizing the origin of these mediators and understanding the ERM-driven neuroinflammation at the vitreal chamber. As reported above, key components of the inflamed retina are Müller cells, astrocytes and microglia/macrophages, which, in concert with floating or entrapped hyalocytes, play significant roles in the (neuro)inflammation at the vitreoretinal interface [33,38,42,45,49,50]. By using a method to select hyalocytes from glial cells inside ERM-collected vitreous samples, we observed that, inside the vitreal pellet, hyalocytes significantly expressed Aβ1-42, TAU and FTH1 in comparison with other cell types. No significant changes were observed for Reelin expression between the two phenotypes (hyalocytes versus vitreal cells) in contrast with GFAP, which, as expected, was significantly expressed by glial cells depauperated of hyalocytes [51]. Hyalocytes and microglia are retinal immune derived cells, acting as sensors of inflammatory stimuli, and become primed by hyperinflammation, which increases the risk of eliciting an exaggerated immune response to autocrine/paracrine secondary inflammatory stimulation with consequent extensive microglial proliferation, toxic debris phagocytosis, anti-inflammatory signals and possible cell cycle block (senescence pathway) [51]. As observed, hyalocytes and microglia provide an important source of pro-inflammatory signals at early ERM stages and, in case of long-lasting contraction (stimulation) that might lead to fibrosis, these cells can increase their own production of pro-inflammatory cytokines and neurotoxic agents (IL6, tumor necrosis factor alpha (TNF), nitric oxide (NO) and reactive oxygen species (ROS) products), depleting the anti-inflammatory ones (IL10 and IL12) [21,47,50]. During neurodegeneration, this pro-inflammatory (IL1β, IL6 and TNFα) enhancement by retinal astrocytes and microglia has been implicated by the release of NO and ROS, causing RGC death and thinning of retinal nerve fiber layers [47,51,52]. Generally, astrocyte and microglia tasks protect RGCs from the effects of oxidative stress and are supplemented with growth factors released by Müller cells [48]. In this context of protecting retinal cells from oxidative damage, FTH1 functions by sequestering redox-active metals (copper, zinc and iron), maintaining iron homeostasis and modulating local inflammation [49,53]. The role of FTH1 in retinogenesis, retinal neurodegeneration and neurodegenerative diseases such as AD has been previously described, prompting us to also investigate this FTH1 that might disrupt iron homeostasis inside healthy retinas, leading to retinal degeneration due to iron toxicity, while mutations in FTH1 have been associated with several neurodegenerative diseases [28,29,53]. Our molecular data indicated that FTH1 is highly produced at stage 3 ERM, in line with the other markers, and is mainly of hyalocyte derivation but can be also produced by astrocytes stimulated by a stage 3 protein profile (vitreous).

Finally, the in vitro studies allowed indirectly to better understand the contribution of retinal astrocytes in the protein signature of ERM vitreous samples. Particularly, an increased transcription of GFAP, Reelin, Aβ1-42, TAU and FTH1 was observed when primary cultures of retinal astrocytes were exposed to stage 3 ERM-derived vitreous samples, while a reduced expression was monitored when stimulating with stage 2 ERM-derived vitreous samples [52]. This finding suggests that the activation of astrocytes and Müller cells can be detrimental or beneficial, as this activation depends on the disease-related protein profile as it can modulate the immune system [51,52,53,54,55,56].

**Figure 6:**
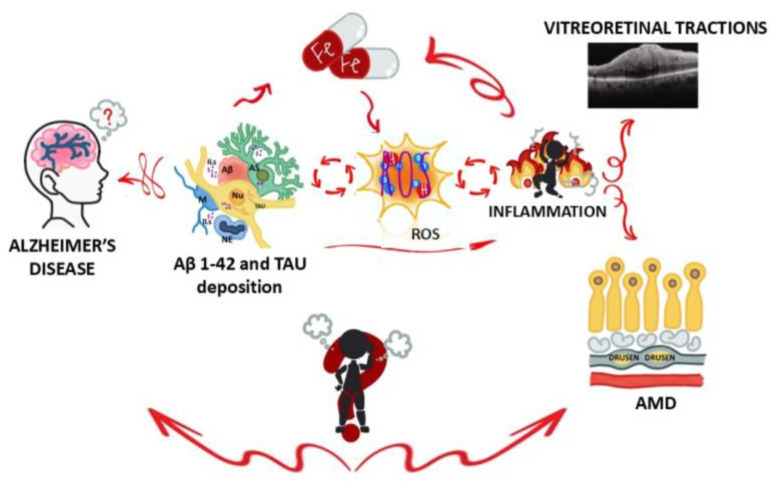
Graphical illustration of inflammation loop during Alzheimer’s disease. A representative image showing how brain and eye (retina) are involved in Alzheimer’s disease (a neurodegenerative disease) and some ocular conditions (vitreoretinal traction and AMD). As stated, β-amyloid accumulation (mainly Aβ1-42 inside soluble and insoluble plaques) and neurofibrillary tangles (chiefly the hyperphosphorylated form) result in a substantial synaptic and neuronal loss [45]. In AD brains, activated microglia and reactive astrocytes are associated with plaques and tangles, so their activation in retinal tissues might be of great relevance for understanding the neurodegenerative microenvironment [13]. The concept behind “burning person” is that the mechanisms of inflammation and oxidative stress that occur in the brain may have similarities or effects in the retina. Legend: Aβ—beta-amyloid; TAU—microtubule-associated protein TAU (tangles); ILs—interleukins; Fe—iron; ROS—reactive oxygen species indicative of oxidative stress; AMD—age-related macular degeneration; NU—neutrophil; M—microglia; AS—astrocyte; NU—neuron; AD—Alzheimer’s disease.

It has been observed that neuroinflammation and neuroprotection run together, as some accessory cells can develop strategies to slow down or prevent neuronal loss (cellular resilience and selected promotion of self-healing) [12,13,14,56,57]. Reelin keeps nerve cells fit, protects neurons from damage and stimulates growth of new neurons (neurogenesis) [58,59,60,61,62]. Reelin has been implicated in APP/Aβ protein processing and regulation of TAU phosphorylation, as observed in AD and Parkinson’s disease [63,64,65,66,67,68,69]. New algorithms supported by the biomolecular database have been recently provided with a great deal of information about retina, brain and early biomarkers, prospecting an alternative and comprehensive method for early diagnosis with easier accessibility, as herein prospected [35,70,71] (Figure 6).

Taken together, this is the first time that a study explores the relationship between Reelin, Aβ1-42, FTH1 and TAU in a vitreoretinal disorder; however, the association of some of those proteins has been previously described, highlighting that lower Reelin levels can exacerbate plaque formation and that β-APP can impair Reelin expression [68,69,70,71]. Reelin production and cleavage, as well as the expression of Aβ1-42, FTH1 and TAU, have been observed in tissues of neuronal derivation when exposed to inflammation, and their dysregulation is more likely to develop neurodegenerative effects (such as in AMD and AD) and warrant further examination [3,58]. Particularly, our findings highlight the association between Reelin and these AD-associated but not exclusive markers in at least ERM tissues, suggesting new alternative diagnostic tools not restricted to the visual system and potential therapeutic targets for counteracting neurodegeneration.

Two main limitations can be identified in this study: First, the small sample size of the study population and second the lack of patients diagnosed with AD. Despite these points, our findings support a possible link between retina and neurodegenerative diseases based on the relation between Reelin, Aβ1-42, FTH1 and TAU. Different immunomodulatory strategies are currently under investigation in preclinical AD models for Reelin potential therapeutic impact in reducing amyloidosis and regulating neuroinflammation [59,60,61,62,72,73,74,75,76].

## 5. Conclusions

Recent studies point out the importance of AD hallmarks common to cerebral and retinal tissues. As retinal tissue may reflect pathological changes occurring in the brain during AD progression, the eye might be considered a very promising structure in the search for new AD biomarkers. The possibility of early diagnosis of AD pathology based on the analysis of Reelin, Aβ1-42, FTH1 and TAU expression in vitreal fluids can be prospected as a starting point for promising diagnostic and therapeutic approaches. Further analyses are ongoing to confirm this association in a larger study population, including a group of patients diagnosed with AD-ERM.

## Figures and Tables

**Table 1:** Primer description.

Genes	Primer Sequence		Genebank (AN)
*Reference gene*			
H3	5′-GCTTCGAGAGATTCGTCGTT	3′-GAAACCTCAGGTCGGTTTTG	NM_005324
*Target genes*			
RELN	5′-GGCATCTTGTCACCGAAGAG	3′-CATTATCAATCGCCCAGGAA	U79716.1
IBA1	5′-GCTGAGCTATGAGCCAAACC	3′-TCGCCATTTCCATTAAGGTC	D86438.1
GFAP	5′-CCCAGCAACTCCAACTAACAAG	3′-ACTCAAAGGCACAGTTCCCA	BC013596.1
CD11b	5′-ACAGAGCTGCCTCTCGGTGGCCA	3′-TTCCC1TCTGCCGGAGAGGCTACC	NM_000632
Aβ1–42	5′-GCCCTTCTCGTTCCTGACAA	3′-GTCATCCTCCTCCGCATCAG	BC004369.1
TAU	5′-ACCATGCACCAAGACCAAGA	3′-TCCAGTCCCGTCTTTGCTTT	BC000558.2
FTH1	5′- GAGGTGGCCGAATCTTCCT	3′-ATGGCTTTCACCTGCTCATTC	M11146.1

Accession numbers (AN; Genebank) were reported by NCBI search, and amplicon length ranged between 100 and 250 bps. Amplification procedure: initial hot start activation (95 °C/5 min) followed by 39 cycles of denaturation (94 °C/10 s)/annealing (58–61 °C/15 s)/extension (75 °C/10 s) and melting curve generation (58–95 °C with one fluorescence reading every 0.5 °C). Legend: AN—accession number.

## Data Availability

All data generated or analyzed during this study are included in this published article.

## Abbreviations

The following abbreviations are used in this manuscript:

AD	Alzheimer’s disease
VLDLR	Very low density lipoprotein receptor
IL-1β	Interleukin-1 beta
ERM	Epiretinal membrane
VEGF	Vascular endothelial growth factor
IOP	Intraocular pressure
Spectralis SD-OCT	Spectral domain optical coherence tomography
PBS	Phosphate-buffered saline
BSA	Bovine serum albumin
FBS	Fetal bovine serum
HBSS	Hank’s balanced sodium solution
IntDen	Integrated optical density
AMD	Age-related macular degeneration
FTH1	Ferritin
GFAP	Glial fibrillary acidic protein
RELN	Reelin
CNS	Central nervous system
RGC	Retinal ganglion cells
TNF	Tumor necrosis factor alpha
NO	Nitric oxide
ROS	Reactive oxygen species
HIF-1	Hypoxia inducible factor-1
MIP-2	Macrophage inflammatory protein 2
